# Implementing parasite genotyping into national surveillance frameworks: feedback from control programmes and researchers in the Asia–Pacific region

**DOI:** 10.1186/s12936-020-03330-5

**Published:** 2020-07-27

**Authors:** Rintis Noviyanti, Olivo Miotto, Alyssa Barry, Jutta Marfurt, Sasha Siegel, Nguyen Thuy-Nhien, Huynh Hong Quang, Nancy Dian Anggraeni, Ferdinand Laihad, Yaobao Liu, Maria Endang Sumiwi, Hidayat Trimarsanto, Farah Coutrier, Nadia Fadila, Najia Ghanchi, Fatema Tuj Johora, Agatha Mia Puspitasari, Livingstone Tavul, Leily Trianty, Retno Ayu Setya Utami, Duoquan Wang, Kesang Wangchuck, Ric N. Price, Sarah Auburn

**Affiliations:** 1 Eijkman Institute for Molecular Biology, Jakarta, Indonesia; 2 Mahidol‐Oxford Tropical Medicine Research Unit, Mahidol University, Bangkok, Thailand; 3 Wellcome Sanger Institute, Hinxton, Cambridge, UK; 4 Centre for Genomics and Global Health, Big Data Institute, University of Oxford, Oxford, UK; 5 School of Medicine, Deakin University, Geelong, VIC Australia; 6 Burnet Institute, Melbourne, VIC Australia; 7 Population Health and Immunity Division, The Walter and Eliza Hall Institute of Medical Research, Parkville, VIC Australia; 8 Department of Medical Biology, The University of Melbourne, Parkville, VIC Australia; 9 Global and Tropical Health Division, Menzies School of Health Research and Charles Darwin University, Darwin, NT Australia; 10 Centre for Tropical Medicine, Oxford University Clinical Research Unit, Ho Chi Minh City, Vietnam; 11 Institute of Malariology, Parasitology and Entomology, Quy Nhon, Vietnam; 12 National Malaria Control Programme, Ministry of Health, Jakarta, Indonesia; 13 UNICEF Indonesia Country Office, Jakarta, Indonesia; 14 National Health Commission Key Laboratory of Parasitic Disease Control and Prevention, Jiangsu Provincial Key Laboratory on Parasite and Vector Control Technology, Jiangsu Institute of Parasitic Diseases, Wuxi, Jiangsu Province China; 15 Pathology, Aga Khan University Hospital, Karachi, Pakistan; 16 Infectious Diseases Division, International Centre for Diarrheal Diseases Research, Bangladesh Mohakhali, Dhaka, Bangladesh; 17 Papua New Guinea Institute of Medical Research, Madang, Papua New Guinea; 18 National Institute of Parasitic Diseases, Chinese Center for Disease Control and Prevention, Shanghai, China; 19 Royal Center for Disease Control, Department of Public Health, Ministry of Health, Thimphu, Bhutan; 20 Centre for Tropical Medicine and Global Health, Nuffield Department of Medicine, University of Oxford, Oxford, UK

**Keywords:** *Plasmodium falciparum*, *Plasmodium vivax*, Malaria, Surveillance, Molecular surveillance, Genotyping, Genomics, SNP barcode

## Abstract

The Asia–Pacific region faces formidable challenges in achieving malaria elimination by the proposed target in 2030. Molecular surveillance of *Plasmodium* parasites can provide important information on malaria transmission and adaptation, which can inform national malaria control programmes (NMCPs) in decision-making processes. In November 2019 a parasite genotyping workshop was held in Jakarta, Indonesia, to review molecular approaches for parasite surveillance and explore ways in which these tools can be integrated into public health systems and inform policy. The meeting was attended by 70 participants from 8 malaria-endemic countries and partners of the Asia Pacific Malaria Elimination Network. The participants acknowledged the utility of multiple use cases for parasite genotyping including: quantifying the prevalence of drug resistant parasites, predicting risks of treatment failure, identifying major routes and reservoirs of infection, monitoring imported malaria and its contribution to local transmission, characterizing the origins and dynamics of malaria outbreaks, and estimating the frequency of *Plasmodium vivax* relapses. However, the priority of each use case varies with different endemic settings. Although a one-size-fits-all approach to molecular surveillance is unlikely to be applicable across the Asia–Pacific region, consensus on the spectrum of added-value activities will help support data sharing across national boundaries. Knowledge exchange is needed to establish local expertise in different laboratory-based methodologies and bioinformatics processes. Collaborative research involving local and international teams will help maximize the impact of analytical outputs on the operational needs of NMCPs. Research is also needed to explore the cost-effectiveness of genetic epidemiology for different use cases to help to leverage funding for wide-scale implementation. Engagement between NMCPs and local researchers will be critical throughout this process.

## Background

Malaria remains a major public health burden in the Asia–Pacific region, with an estimated 10 million cases and 15 thousand deaths in 2018 [1]. Nonetheless, there has been a substantial decline in malaria cases over the last 2 decades, that has enabled national malaria control programmes to start implementing strategies for malaria elimination. In 2009, the Asia Pacific Malaria Elimination Network (APMEN) was established to address the unique challenges of malaria elimination in the region, through knowledge exchange, capacity strengthening and building the evidence base for elimination [2, 3]. Several years later, the Asia Pacific Leaders Malaria Alliance (APLMA) was formed to enhance and streamline regional response to malaria, with the goal of eliminating this disease from the region by 2030. However, the challenges to achieve this goal are considerable.

The revolution in genomic technologies has created new opportunities to study how malaria parasites adapt and spread within and across borders and has the potential to help answer key epidemiological questions that could help NMCPs form better elimination strategies. However, there are currently significant obstacles preventing wide adoption of molecular technologies to endemic countries and their integration into national public health systems. Recent reviews have discussed the challenges of integrating genomic surveillance for malaria in Africa [4–6]; similarly, the authors sought to dissect the challenges hindering the adoption of genomic technology in the Asia–Pacific region. Although the burden of malaria in the Asia–Pacific region is considerably lower than that in Africa, the region faces additional challenges of a high proportion of non-falciparum malaria, as well as being the epicentre of the emergence of resistance against frontline anti-malarials, such as artemisinin and its partner drugs [7–10].

In November 2019, the Menzies School of Health Research (Menzies), in collaboration with the Eijkman Institute for Molecular Biology (Eijkman), conducted a two-day parasite genotyping workshop in Jakarta, Indonesia. The concept for the workshop stemmed from discussions within APMEN around the need to address the challenges of parasite genetic surveillance in the region. As a result, APMEN participants from 21 countries, as well as several self-funded collaborators were invited to join the 2 day workshop, which was co-funded by APMEN, the Australian Department of Foreign Affairs and Trade (DFAT) and the Australian Center of Research Excellence in Malaria Elimination (ACREME). The meeting was attended by 70 participants, with representatives from 8 malaria-endemic countries in the Asia–Pacific region; Bhutan, Bangladesh, Pakistan, Thailand, Vietnam, China, Indonesia and Papua New Guinea. Participants included representatives of NMCPs, researchers, United Nations Children's Fund (UNICEF) and the World Health Organization (WHO). The aim of the workshop was to review recent advances in molecular approaches for *Plasmodium falciparum* and *Plasmodium vivax* parasite surveillance and explore ways in which novel surveillance strategies could be integrated into policy and practice.

This reports key messages, with specific focus on the following topics:

I. Use cases for parasite genotyping in surveillance.
II. Technical challenges in implementing genotyping in-country.
III. Data sharing within and between countries.
IV. Maximizing the value of parasite genotyping for NMCPs.

### Use cases for parasite genotyping in surveillance

Dr Sarah Auburn, from Menzies, gave an overview of use cases for genetic epidemiology that support the operational needs of NMCPs [11], and led discussions centred around assessing their local utility, as well as identifying new relevant use cases.

#### Detect resistance

Following the widespread failure of chloroquine and sulfadoxine-pyrimethamine (SP) treatments against *P. falciparum*, the implementation of artemisinin-based combination therapy (ACT) in the early-to-mid 2000s has been a major contributor to the remarkable success in case reductions in the Asia–Pacific region [1]. Therefore, the emergence and spread of *P. falciparum* strains resistant to artemisinin and some of its ACT partner drugs in the Greater Mekong subregion (GMS) is a major concern that has elicited an emergency response to contain further spread. Clinical, laboratory and molecular studies have identified that mutations in the *k13* gene located on chromosome 13 of *P. falciparum* determine and modulate resistance to artemisinin [12–14]. Although multiple *k13* mutations have been associated with artemisinin resistance, the C580Y mutation has become dominant in the GMS in the last few years [15]. Mutations associated with resistance to ACT partner drugs have become widespread, including copy number amplifications of the *plasmepsin 2/3* genes (*pfpm2/pfpm3*) and the *multidrug resistance 1* gene (*pfmdr1*), which confer resistance to piperaquine and mefloquine respectively [16–18]. Genetic correlates of parasite resistance to several commonly used anti-malarials in the Asia–Pacific region are summarized in Table 1. The malaria Genomic Epidemiology Network (malariaGEN) community has developed a set of rules for classifying parasites in terms of drug resistance, based on the alleles detected at these markers [19].

**Table 1:** Summary of genetic correlates of drug resistance in *P. falciparum* and *P. vivax*

Species	Anti-malarial	Country (policy)^a^	Gene	Validated mutations	Associated mutations	References
*P. falciparum*	Artemisinin derivatives	BD, BT, KH, CN, ID, IN, LA, MY, MM, NP, PH, PG, SB, TH, TL, VU, VN	*pfkelch13*	Y493H, R539T, I543T, R561H, C580Y	P441L, F446I, G449A, N458Y, M476I, N537D, P553L, V568G, P574L, M579I, D584V, A675V, H719N	[12, 13]
	Piperaquine	CN, ID, MM, TH, VN	*pfpm2/pfpm3*	≥ 2 copies of the gene	–	[16, 17]
	Mefloquine	KH, MY, MM	*pfmdr1*	≥ 2 copies of the gene	S1034C, N1042D	[18, 83, 84]
	Lumefantrine	BD, BT, CN, ID, LA, MM, NP, PH, PG, SB., TL, VU	*pfmdr1*	–	N86Y,	[85, 86]
	Chloroquine	ID, KR, SB (IPT), VU (IPT)	*pfcrt*	K76T with; M74I and N75E, or C72S	–	[87]
			*pfmdr1*	–	N86Y, S1034C, N1042D, D1246Y	[84, 88]
	Sulfadoxine	ID, PG (IPT)	*pfdhps*	S436A, K437G, K540E, A581G, A613S/T	–	[89, 90]
	Pyrimethamine	PG (IPT)	*pfdhfr*	C50R, N51I, C59R, S108N, I164L	–	[89]
	Amodiaquine	CN	*pfcrt*	C72S and K76T	–	[91]
			*pfmdr1*	–	N86Y, D1246Y	[81, 91]
	Pyronaridine	CN	–	–	–	–
	Primaquine	ID, IN, LA, NP, PH	–	–	–	–
	Quinine	BD, BT, KH, ID, IN, MY, MM, PH, KR, SB, TL, VN	–	–	–	–
*P. vivax*	Chloroquine	BD, BT, CN, KP, ID, MM, NP, PH, KR, SL, TH, VN	*pvcrt-o*	–	Increased expression, 14 TGAAGH motifs in intron 9	[25, 26]
			*Intergenic*	–	15 TGAAGH motifs at MS334 (upstream of *pvcrt*-o)	[26]
			*pvmdr1*	–	Increased expression, Y976F, F1076L	[25, 92, 93]
	Sulfadoxine	–	*pvdhps*	–	A383G, A553G	[94, 95]
	Pyrimethamine	–	*pvdhfr*	–	F57L, S58R, T61M, S117T, S117N	[96–101]
	Mefloquine	KH, MY	*pvmdr1*	–	≥ 2 copies of the gene	[102–104]
	Amodiaquine	–	*pvmdr1*	–	Y976F	[96]
	Artemisinin derivatives	KH, IN, LA, MY, PH, PNG, SB, TL, VU	–	–	–	–
	Piperaquine	CN, IN	–	–	–	–
	Lumefantrine	LA, MY, PH, PG, SB, TL, VU	–	–	–	–
	Primaquine	BD, BT, KH, CN, KP, ID, IN, LA, MY, MM, NP, PH, PG, KR, SB, TH, TL, VU, VN	–	–	–	–

Countries are listed using two letter codes *BD* Bangladesh, *BT* Bhutan, *KH *Cambodia, *CN* China, *IN* India, *ID* Indonesia, *KP* DPR of Korea, *KR* Rep. of Korea, *LA* Lao PDR, *MY* Malaysia, *MM *Myanmar, *NP* Nepal, *PH* Philippines, *PG* PNG, *SB* Solomon Is., *TH* Thailand, *TL* Timor-Leste, *VN* Vietnam, *VU* Vanuatu

^a^Countries in the Asia–Pacific region where the given anti-malarial drug is implemented as national drug policy (alone or in combination) for treatment of uncomplicated unconfirmed, uncomplicated confirmed or severe *P. falciparum* or *P. vivax* infection, or for intermittent preventative treatment in pregnant women (IPT) [1]

Surveillance of *P. falciparum* drug resistance was raised as one of the highest priorities of participants from high and intermediate transmission regions in the GMS and other areas. Until recently, there was no convincing evidence of artemisinin resistance in the Pacific region. Olivo Miotto from the Mahidol Oxford Research Unit (MORU), Thailand, described the recent detection of three *P. falciparum* infections in Wewak, Papua New Guinea (PNG) [118], which carried C580Y mutations on a genetic background distinct from that observed in the GMS, highlighting the importance of vigilance across the region. Alyssa Barry from Deakin University, Australia, presented data on behalf of STRIVE (Stronger Surveillance and Systems Support for Rapid Identification and Containment of Resurgent or Resistant Vector Borne Pathogens) PNG, an international team including the PNG NMCP and PNG Institute of Medical Research, showing that these mutations had not yet spread to other parts of the country, and no clinical resistance has been detected.

Molecular surveillance of determinants of anti-malarial drug resistance was acknowledged as an important early warning system to identify hotspots of resistance, prioritizing where therapeutic efficacy surveys should be conducted, and informing on which alternative treatment regimens could be effective in areas with high levels of failures against the current frontline treatment. Nguyen Thuy-Nhien from the Oxford University Clinical Research Unit (OUCRU), Vietnam, described large-scale molecular surveillance at 50 sites in 9 provinces of Vietnam, implemented as part of the GenRe-Mekong project in close collaboration with the NMCP. This implementation leverages on SpotMalaria (https://www.malariagen.net/projects/spotmalaria), a technology framework providing high-throughput genotyping of malaria parasites at a broad selection of single nucleotide polymorphisms (SNPs). SpotMalaria genotypes all *k13* mutations, *pfpm2/pfpm3* amplifications, and most important markers of resistance to current and historical drugs, as well as a genetic barcode of 101 neutral SNPs and variants informative of co-infecting species. Since the project’s establishment in 2017, information provided by GenRe-Mekong, in collation with clinical data, has contributed to anti-malarial drug policy changes in two provinces in Vietnam, underscoring the great translational utility of this genotyping use case.

In many Asia–Pacific countries, chloroquine is still the recommended first-line treatment for blood stage *P. vivax*. In Cambodia, Indonesia, Malaysia, PNG, Vanuatu and Solomon Islands, high rates of chloroquine failure against *P. vivax* have led to a policy change towards ACT as first-line treatment for all species of malaria [20–22]. Jutta Marfurt, from Menzies, gave an overview of anti-malarial drug resistance in *P. vivax*, emphasizing the lack of validated molecular markers of clinical chloroquine or artemisinin resistance in this parasite (Table 1).

Although the Y976F mutation in the *P. vivax multidrug resistance 1* gene (*pvmdr1*) is frequently genotyped in research studies, this variant is a minor modulator of chloroquine sensitivity and, therefore, not informative in the absence of phenotypic data from clinical or laboratory studies [23]. The role of the *P. vivax chloroquine resistance transporter* gene (*pvcrt-o*) also remains unclear, with conflicting patterns of association between *pvcrt-o* expression and chloroquine susceptibility observed in field studies [24, 25]. A repeat-length polymorphism associated to changes in *pvcrt-o* expression has recently been identified in a laboratory-based study, but further evaluation is needed in patients with clinical disease before it is used as a marker of chloroquine resistance [26]. The absence of molecular markers for *P. vivax* remains a major gap in molecular surveillance frameworks.

#### Assess drug resistance gene flow

Identifying reservoirs of drug resistant strains, and the routes through which they spread, within and across borders, can help NMCPs to map the geographical areas at greatest risk of treatment failures, and plan suitable interventions and alternative treatment strategies. Genetic data on malaria parasites can provide deep insights on the origins and genetic make-up of drug resistant variants, and the lineages that carry them. Combining newly generated and publicly available whole genome sequencing data, Miotto and colleagues were able to demonstrate that the three PNG C580Y mutants in Wewak were not imported from the GMS; rather, they shared close genetic relatedness to Papua Indonesian isolates and carried a C580Y mutation that has arisen independently [118]. An alternative approach to whole genome sequencing is genotyping highly polymorphic markers in the regions flanking the drug resistance variant(s); for example, Imwong and colleagues used flanking microsatellites to reconstruct the routes of spread of a C580Y mutant lineage in the GMS [27]. However, aligning microsatellite-based datasets generated by different groups can be difficult, and artefacts such as stutter can be problematic for reliable genotype calling [28]. An informative SNP panel needs to be developed to enable a high-throughput approach for monitoring gene flow.

#### Assess transmission intensity

Determining transmission intensity is critical for NMCPs to monitor progress and optimize intervention strategies in different areas; for instance, to identify where to focus resources for interrupting residual transmission and minimizing the risk of imported parasites in areas close to malaria elimination. Traditional surveillance methods incorporating tools such as microscopy and rapid diagnostic tests (RDTs) can gauge the prevalence or incidence of infections but rely on local treatment-seeking behaviour and diagnostic accuracy. Amplification of parasite DNA, such as Polymerase Chain Reaction (PCR), improves diagnostic sensitivity but is still reliant on effective methods for detecting infected individuals. Parasite genotyping has been proposed as a complementary method to provide information on the parasite population dynamics in a given area, by estimating indices of transmission intensity from genetic data. In some endemic settings, the frequency of polyclonal infections or complexity of infection (COI) are good correlates of transmission intensity [29], since polyclonal infections are more commonly observed in high transmission areas, where more infected mosquitoes provide greater opportunity for superinfection. However, such relationships are not necessarily observed in all settings, possibly because of imported infections [30, 31], differences in sampling methods [32], or differing marker sets or genotype calling methods. Several statistical methods are available to estimate polyclonality: for instance, Multiplicity of Infection (MOI) is often applied to microsatellite-based datasets, within-sample fixation index (*F*_WS_) to genomic SNP datasets, and the likelihood-based COIL method for measuring COI using SNP barcodes [28, 33, 34]. Several genotyping platforms are available for assessing COI. Traditionally, parasite genotyping has used PCR to amplify gene regions with repeat-length polymorphisms, such as the *P. falciparum* merozoite surface proteins 1 and 2 (*pfmsp1* and *pfmsp2*) and orthologous *P. vivax* gene families or microsatellites [35–39]. Multiple clone infections can be detected by the presence of multiple PCR amplicons of differing lengths as detected by investigation of band patterns on agarose gels or capillary sequencing. In recent years, deep sequencing of a targeted selection of highly diverse gene regions using amplicon sequencing on massively parallel sequencing platforms such as Illumina, has become the favoured approach to characterize the complexity of malaria infections [40]. The extensive depth of sequence reads generated by amplicon deep sequencing (100–1000 s of reads per target gene depending on the sequencing conditions) enables substantially greater sensitivity to detect minor clones than capillary sequencing. Studies in PNG, where transmission is high and infections are frequently complex, have demonstrated the detection of minor *P. falciparum* clones at a detection limit of 1:1000, and used these methods to track longitudinal infection dynamics with high sensitivity [41–43]. Amplicon deep sequencing has also been used in *P. vivax* to compare infection dynamics between day 0 and recurrent infections [44]. Several analysis tools have been developed to support the analysis of infection complexity using deep sequencing data, including PASEC, DADA2, HaplotypR and SeekDeep [43, 45–47]. Despite differences in their approach, these four state-of-the-art tools resolved known haplotype mixtures with similar sensitivity and precision [47].

The study of genetic relatedness and population structure can also be informative of transmission levels: studies of both *P. falciparum* and *P. vivax* have observed increasing genetic relatedness between infections as parasite populations dwindle, as inbreeding and clonal transmission becomes predominant [48–53]. Shrinking populations tend to form moderately distinct pockets (or foci) of infection, which translate to changes in population structure, often modulated by external factors. In contrast to conventional methods such as microscopy or RDTs, genetic indices require representative sampling, but are not reliant on comprehensive sampling, which makes them potentially robust tools for assessing transmission intensity.

An example of the use of endemicity data by NMCPs was provided by Nancy Dian Angraenni from the Ministry of Health, Indonesia. She outlined the “island by island” malaria elimination strategy in Indonesia, where areas are categorized according to the local endemicity, and control activities are applied in stages from the regency/city administrative levels to the provincial and then regional and national levels. These efforts require detailed maps of malaria risk to guide the strategic distribution of limited resources, with regular updates as control progresses [54]. A better understanding of how genetic indices correlate with malaria elimination categories will help to stratify interventions in areas where regular and comprehensive surveillance by conventional methods is not feasible.

#### Identify foci of infection

This use case defines target areas for tailored interventions; examples include localizing hotspots of transmission in heterogenous regions, or residual transmission foci in low endemic areas. This is not a high priority in high transmission areas, where infections tend to be uniformly distributed and distinct foci of infection are generally rare [55]. However, as transmission declines and parasite populations shrink, distinct foci of infection begin to emerge. These are currently identified from geospatial surveillance and case investigations, but detection can be improved by comparing the parasite genetic relatedness within versus between foci [11]. Further research is needed to determine operationally relevant spatial scales for defining the boundaries of foci, and the stability of these foci over time.

#### Determine connectedness between parasite populations

Estimating the connectedness between parasite populations can help gauge the risks of parasites spreading between geographic areas. This information is particularly important for locating major reservoirs (“sources”) that are sustaining infections in other areas (“sinks”). Connectedness can be estimated using human mobility data, such as patient travel history or mobile phone data, but two recent studies highlight the potential of combining these data with parasite genetic data, to reveal both local and long-distance parasite transmission routes [56, 57]. In contrast to mobile phone data, which provide information about movement of the human population, genetic data offer a view on parasite gene flow, whose routes may differ from those followed by people. Furthermore, genetic data is not restricted to the coverage of mobile networks, which is particularly valuable across country borders [56, 57].

A classical approach used to quantify connectedness between populations is the assessment of genetic differentiation using the fixation index (*F*_ST_) [58]. This measure has been used widely in microsatellite-based studies of *P. falciparum* and *P. vivax*, providing useful insights on the connectedness between populations at a moderately granular scale [59]. However, *F*_ST_ does not account for recombination, and is constrained in its ability to infer fine-resolution connectedness [60]. Conversely, measures of identity by descent (IBD) apply a probabilistic model accounting for recombination and can provide insights into more recent demographic changes. IBD is increasingly being used to assess parasite connectedness at relatively small spatial scales [60–62]. Tools for measuring IBD include isoRELATE, hmmIBD and DEploid-IBD [61–63].

Low-resolution genetic studies of co-endemic *P. falciparum* and *P. vivax* in Indonesia and PNG have demonstrated higher connectedness amongst *P. vivax* populations, highlighting the greater potential for this species to spread via the hypnozoite reservoir [64, 65]. Rintis Noviyanti, from Eijkman, described plans to expand on the maps of parasite connectedness across Indonesia using high-throughput SNP-based genotyping methods. Alyssa Barry and Livingstone Tavul, from the PNG Institute for Medical Research, described similar plans for PNG. The evidence generated from these studies will be shared with the local NMCPs, with a view to implementing them more widely within local surveillance frameworks.

#### Identify imported cases and transmission chains

**Fig. 1:**
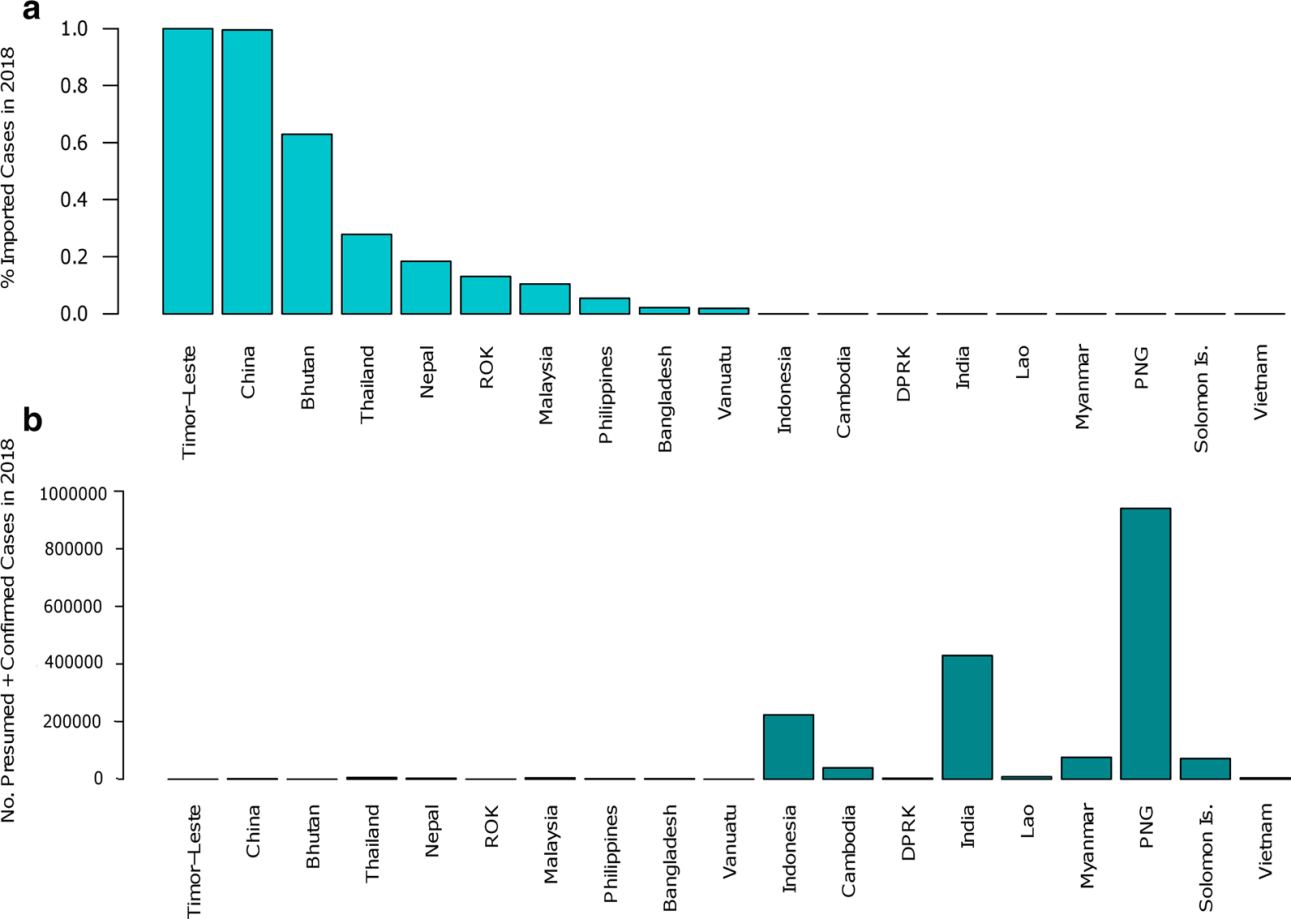
Imported case proportions and total malaria case numbers in the Asia–Pacific region in 2018. **a** presents the percentage of imported cases (all species of malaria) in 2018 in order of highest to lowest percentage. **b** presents the total number of presumed and confirmed cases (for all species) in 2018 in the same order as (**a**). The numbers were derived from the World Malaria Report 2019 summary of reported malaria cases by method of confirmation 2010–2018 for countries in the WHO Southeast Asia and Western Pacific region. The percentage of imported cases was calculated as the number of imported cases divided by the total number of presumed and confirmed cases. The countries with the highest proportion of imported cases have amongst the lowest number of overall cases

In low-endemic areas of the Asia–Pacific region, border malaria and imported cases pose major challenges to malaria elimination, particularly in areas with highly mobile human populations. Figure 1 illustrates the relative proportions of autochthonous versus imported infections in different countries in the region. The meeting agreed on the importance of distinguishing between border and imported malaria, since these reflect distinct challenges with different surveillance and response needs. Duoquan Wang from the National Institute of Parasitic Diseases at the Chinese Centre for Disease Control, and Yaobao Liu from the Jiangsu Institute for Parasitic Diseases, China, described China’s remarkable success in reducing indigenous malaria cases from 24 million in 1970 to zero since 2017 [1], and highlighted post-elimination surveillance of border and imported malaria as one of the highest priorities for maintaining a malaria-free status. Liu described the malaria situation in Jiangsu province of central China [66], and the broader situation in China, describing how most reported infections in the past few years were suspected importations from Africa (~ 80% *P. falciparum*) and southeast Asia (~ 80% *P. vivax*) [67]. In several high transmission countries, there is interest in tools for surveying imported cases of *P. falciparum* from regions such as the GMS where artemisinin-resistance is prevalent.

At present, imported cases are usually identified and mapped using information on patient travel history. However, the procedure followed has been designed specifically for *P. falciparum* malaria, without consideration for the delayed relapses of *P. vivax*, which may lead to incorrect conclusions. Molecular tools to identify and map imported malaria cases offer a complementary approach to traditional epidemiology. Table 2 summarizes different molecular approaches that have been used to map imported *P. falciparum* and *P. vivax* cases. Hidayat Trimarsanto described a collaborative project between Eijkman and global partners within the vivax Genomic Epidemiology Network (vivaxGEN), to develop molecular tools for identifying and mapping imported *P. vivax* cases. Investigation of genomic data from 831 *P. vivax* genomes from 20 countries identified 28 new genetic markers that can be used to distinguish imported from local infections [68]. A web-based tool, vivaxGEN-GEO, was developed to map country of origin, even in the presence of missing or heterozygous genotype calls (https://geo.vivaxgen.org/).

**Table 2:** Molecular methods to identify and map the geographic origin of malaria infections

Markers	Species	Platform^a^	Assays	Comments	Publications
Variable surface antigens: *pvcsp and pvmsp1*	*P. vivax*	Capillary sequencing	Primers available to amplify the gene regions	Diversity may reflect selection from the host immune system, rather than geographic ancestry. Lower resolution than genome-wide SNP barcodes	[105–107]
Microsatellites	*P. vivax*	Capillary sequencing	Primers available to amplify the gene regions of a 9-marker set used by the APMEN vivax Working Group	Microsatellites are often neutral but have high mutation rates constraining geographic ancestry. A single marker (MS20) appears to discriminate temperate from tropical *P. vivax* infections. Lower resolution than genome-wide SNP barcodes	[108, 109]
Mitochondrial genes	*P. falciparum* and *P. vivax*	Capillary sequencing	Primers available to amplify the gene regions	Mitochondrial genome is historically conserved and robust to recombination. Extensive reference datasets available from across the globe in GenBank. Lower resolution than genome-wide SNP barcodes	[110–113]
23-SNP mitochondrial and apicoplast barcode	*P. falciparum*	Targeted SNP genotyping	In silico only (no assays developed to date)	Mitochondrial and apicoplast genomes are historically conserved and robust to recombination. Addition of the apicoplast genome provides more variants, increasing resolution. Robust regional-level resolution	[114]
42-SNP genome-wide barcode	*P. vivax*	Targeted SNP genotyping	Real-time PCR high-resolution melt (HRM) assays. Amplicon sequencing Illumina assays	Less robust to effects of recombination over time than mitochondria and apicoplast. Robust regional-level resolution	[115]
28-SNP, 50-SNP and 51-SNP genome-wide barcodes	*P. vivax*	Targeted SNP genotyping	In silico at present but Illumina amplicon sequencing and minION assays in development	Less robust to effects of recombination over time than mitochondria and apicoplast. Robust country-level resolution in many areas. Online data analysis tool available, amenable to missing data and polyclonal infections: vivaxGEN-GEO (https://geo.vivaxgen.org/)	[68]

^a^See Table 3 for comparative assessments of different genotyping approaches

Duoquan Wang described the challenge of border malaria in Yunnan Province, southern China, which shares hundreds of kilometres of border with Myanmar [69]. Cross-border parasite flow is high along large stretches of the border region, with most cases believed to migrate from Myanmar into China; analogous cross-border flow in Bhutan’s southern districts neighbouring India were reported by Kesang Wangchuk from the Ministry of Health, Bhutan. The challenge is more pronounced for *P. vivax* than *P. falciparum*, as dormant hypnozoites can enable longer persistence of *P. vivax* infections and accordingly wider dispersal. High rates of parasite flow across porous border regions result in relatively homogenous parasite populations in neighbouring countries, hampering classification of local from imported cases using categorical classification-based methods such as vivaxGEN-GEO. Whilst country classifications may be difficult to obtain using genetic data in highly porous border regions, genotyping can potentially be useful to decipher parasite transmission chains; using genetic barcodes to distinguish infections, the transmission of individual parasite strains can be traced from one individual to another. This information can be used to monitor whether cases with travel history from across the border have been transmitted locally. It was agreed by the meeting that genetic homogeneity between neighbouring parasite populations poses significant challenges to monitoring malaria importation and provides a strong case for cross-country collaborative efforts and data sharing.

#### Detect HRP2/3-based rapid diagnostic test failures

*Plasmodium falciparum* infections with deletions of histidine-rich protein 2 and 3 (HRP2 and HRP3) may go undetected by several RDTs [70]. Recent insights from genomic data on *P. falciparum* indicate that up to 25% of infections in Papua Indonesia and PNG have HRP3 deletions [19], suggesting that molecular surveillance of these parasites is needed. However, molecular analysis of HRP2 and HRP3 variants affecting RDT efficacy remains complex. The WHO recommends PCR-based methods to analyse the exons and flanking genes of HRP2 and HRP3, and a 7-point quality scoring system based on recommendations by Cheng and colleagues [71]. To date, there are no high-throughput frameworks for surveillance of these genes.

#### Characterize outbreaks

Countries with a high burden of malaria have a huge challenge in providing robust surveillance systems and thus are often not able to assess geospatial and temporal trends such as outbreaks, undermining optimal responses to malaria control [1]. Several molecular methods are available to investigate outbreaks, using methods similar to those used for detecting transmission chains. Using genetic barcodes to compare infections, it is possible to identify the rapid clonal expansion of specific strains, possibly indicating that they possess some fitness advantage [49]. Concurrent genotyping at relevant drug resistance determinants, geographic markers to identify imported cases and HRP deletions can add additional information to support such investigations.

#### Characterize recurrent *P. vivax* infections

The characterization of recurrent *P. vivax* infections was also deemed to be a genetic use case in the Asia–Pacific region. Relapses deriving from dormant liver-stages (hypnozoites) are the main contributor to the transmission of *P. vivax* infections, as they may be the source of over 80% of all cases [72, 73]. Distinguishing relapses from reinfections is complex, since hypnozoites causing a relapse may be genetically different from the parasites that caused the initial infection [74]. Recent studies using whole genome sequencing data have demonstrated the utility of molecular data to identify recurrent infections that are genetically different from day-zero parasites, but appear to be related, sharing identity by descent [75, 76]. Pairs of day-zero and recurrent parasites that are genetically related are more likely to have derived from the same mosquito bite than from different inoculations (as in a reinfection) and are, therefore, more likely to represent relapse rather than re-infection events. Paired *P. vivax* isolates have been compared using microsatellites [119]. Methods using microhaplotypes (short stretches of the genome which contain multiple SNPs and, therefore, can be treated as a single locus with more than two alleles) are also in development [42]. However, whilst small sets of microsatellites and microhaplotypes can capture genetic similarity due to identity by state (IBS), they may miss pairs of siblings that have high IBD (> 50%) but low IBS because of recombination. Sasha Siegel, from the Wellcome Sanger Institute, described a collaborative project with Menzies to develop a comprehensive panel of approximately 100 microhaplotypes across the *P. vivax* genome, as an alternative to whole genome sequencing to determine which pairs of infections are related based on measures of IBD and, therefore, likely to reflect relapse events. Mathematical models are being developed to analyse this information in surveillance frameworks, to infer the underlying burden of relapses from passively collected pairs of infections. The aim of this approach is to inform on the efficacy of and adherence to primaquine or other radical cure policies.

### Technical challenges in implementing genotyping in-country

Discussions around genotyping use cases highlighted a variety of different needs and priorities for parasite surveillance, largely reflecting the varying endemicity across the Asia–Pacific region. This raised questions about the suitability of a “one size fits all” approach in countries ranging from high burden areas to only handfuls of imported cases (Fig. 1). Each country needs to choose an implementation of genetic surveillance that suits their needs and maximizes the value they can get from the resources they have available. Indeed, the access to specialized molecular technologies varies widely between countries: whilst China is world-renowned for its capabilities, with large sequencing centres providing services to clients across the globe, many other countries in the region have poor access to genomic services.

The varying access to genomic services fuelled discussion around the pros and cons of whole genome sequencing for parasite surveillance. In the pros, whole genome sequencing provides the highest genetic information content on a sample, with the potential to genotype thousands of SNPs, as well as copy number variants (CNVs) and other structural rearrangements.

Genomic data also provides the greatest potential to characterize and disentangle polyclonal infections using methods such as DEploid [63]. With the costs of sequencing continually falling, whole genome sequencing of malaria parasites is becoming increasingly cost-effective. However, the cons of whole genome sequencing include difficulty in obtaining high-quality samples from patient infections, particularly for low-density *P. vivax* infections. Although selective whole genome amplification (sWGA) methods have been established for *P. falciparum* and *P. vivax* to overcome the challenges of low-density infections and high human DNA contamination [77, 78], these methods constrain detection of CNVs, and coverage of the *P. vivax* genome can remain sparse in low-quality samples. The biggest constraints to whole genome sequencing in many parts of the Asia–Pacific are the limited technical and bioinformatic skills required to achieve high-quality genomic data and to handle and analyse the large datasets.

For many of the use cases described, whole genome data is unnecessary; carefully selected subsets of SNPs (SNP barcodes) or other variants can provide the information needed by NMCPs. A range of platforms are available for genotyping SNP barcodes, producing data that is informative but requires smaller investments than whole genome sequencing and is more suitable for entry-level expertise. An example of such an approach is amplicon sequencing, which performs high-throughput sequencing, but only at loci of interest. As a result, it is more cost effective than whole genome sequencing, simpler to analyse, and can still provide a comprehensive profiling of the parasites, by typing drug-resistance mutations as well as genetic barcodes that “summarize” the genome. A technical session was held to discuss the relative pros and cons of amplicon sequencing and other genotyping platforms that are currently being considered for parasite surveillance (see Table 3 for a summary).

**Table 3:** Overview of several genotyping methods used for malaria samples

Method	Marker throughput	Sample throughput	Sensitivity	Test-to-result time	Cost considerations	Accessibility	Sequencing features	Data handling
Capillary sequencing	One gene region at a time	Low to high	Moderate at major allele, low at minor^1^	Days	Not cost-effective for multiple genes in a large sample	Widely accessible. Technical expertise often available in endemic countries	Accessibility to moderately complex sequence regions. Ability to detect new variants and VNTRs^2^. Suitable for genotyping tri- or quadri-allelic positions	Time-consuming to review multiple sequence traces
Microsatellite typing by capillary sequencing	One to ~ four markers at a time	Low to high	Moderate at major allele, low at minor^1^	Days	Not cost-effective for multiple genes in a large sample	Widely accessible. Technical expertise often available in endemic countries	Multi-allelic nature helps to characterize polyclonal infections. Stutter and other artefacts can be problematic	Time-consuming to review multiple sequence traces
SNP genotyping by HRM^3^	One marker at a time	Low to high	Moderate at major allele, low at minor^1^	Days	Not cost-effective for multiple genes in a large sample	Accessible and user-friendly technology	Accuracy in genotyping heterozygote positions is constrained. Need controls for every marker on each run	Time-consuming to review multiple sequence traces
Real-time PCR analysis of CNVs^4^	One gene region at a time	Low to high	Moderate at major allele, low at minor^1^	Days	Not cost-effective for multiple genes in a large sample	Accessible and user-friendly technology	Optimal for CNVs. Need controls for every marker on each run	Time-consuming to review multiple sequence traces
MassARRAY genotyping	One to ~ 40 markers at a time	Moderate to high	Moderate at major, low at minor^1^	Weeks	Cost-effective for moderate-large sample size and multiple genes	Not highly accessible. Requires specialized technical expertise (reference lab advised)	Accuracy in genotyping heterozygote positions is constrained	Need specialized skills
Amplicon sequencing with Illumina, and Molecular Inversion Probes	Dozens to hundreds of markers in parallel	Moderate to high	High at major and minor allele^5^	Weeks^6^	Cost-effective for large sample size and multiple genes	Not highly accessible. Requires specialized technical expertise (reference lab advised)	Digital allele calling. Potential to detect CNVs^4^. Not feasible for detecting new variants	Need specialized skills
MinION genotyping	Dozens to hundreds of markers in parallel	Low to high	Moderate at major, low at minor^1^	Days	Cost-effective for small-moderate sample size and multiple genes	Highly portable, accessible and user-friendly to run	Ability to detect new variants and VNTRs^1^. Accessibility to moderately complex sequence regions. High rate of sequencing errors	Need specialized skills, but amenable to more user-friendly platforms

^1^Generally not robust to detect minor alleles at intensity lower than 10% of major allele. ^2^Variable Number Tandem Repeats. ^3^High Resolution Melt-curve analysis using quantitative PCR. ^4^Copy Number Variants. ^5^Depends in part on read depth, which is partly determined by the multiplexing level. ^6^Depends on sample throughput; turnaround time of weeks assumes a moderately large sample throughput for cost-efficacy

Olivo Miotto gave an overview of the SpotMalaria amplicon sequencing framework and SNP barcodes used by the GenRe-Mekong project to genotype *P. falciparum* and *P. vivax* samples (https://www.malariagen.net/projects/spotmalaria). An overview of the SpotMalaria barcodes and several other *Plasmodium* SNP barcodes is provided in Table 4. Amplicon sequencing uses high-throughput Illumina sequencers to sequence selected loci across the *Plasmodium* genome, enabling multiplexing that allows hundreds of samples to be genotyped in a single experiment, providing cost-effective genotyping of large sample sets with high accuracy and sensitivity. The sensitivity will depend in part on the multiplexing level and other sequencing conditions, but is potentially even amenable to leftover clinical material from RDTs [79]. The platform is flexible to iterative enhancements and extensions of the SNP barcodes. The reliance on specialized equipment and reagents, and the need for highly skilled technicians, limit amplicon sequencing implementations to few laboratories, best achieved through a centralized genotyping framework within a given malaria-endemic country, or regional reference laboratories supporting multiple countries. Networks such as APMEN, ACREME, vivaxGEN and MalariaGEN can support regional efforts, promoting standardization and knowledge sharing. The high-throughput nature of amplicon sequencing lowers the turnaround time for data to be returned, which is typically of the order of weeks or months in current implementations. Although this turnaround is not suitable for supporting point-of-care decisions, it is valuable for use by NMCPs needing to make policy decisions based on population-level data. The two examples of policy changes in Vietnam were supported by population-level genetic data on drug resistance variants (in collation with clinical data), which is provided to the NMCPs (Institute of Malaria, Parasitology and Entomology in Vietnam) on a quarterly basis (approximately every 3 months).

**Table 4:** Overview of several SNP panels for barcoding *P. falciparum* and *P. vivax* infections

Marker panel	Species	Description	Applications	Genotyping platforms	Countries implementing the panel	Publication
^1^SpotMalaria 101 SNPs	*P. falciparum*	101 SNPs including neutral variants, species-specific variants and drug-resistance markers in K13, *pfcrt*, *pfdhfr*, *pfdhps, pfmdr1* and *pfpm2/pfpm3* SNPs and copy number,	Drug resistance prevalence, species confirmation, infection complexity, genetic relatedness	Illumina amplicon sequencing	Bangladesh, Cambodia, Ghana, Benin, Brazil, Cameroon, Colombia, Congo, Ethiopia, The Gambia, Guinea, India, Indonesia, Kenya, Laos, Malaysia, Mali, Myanmar, Peru, Senegal, Sudan, Tanzania, Thailand, Vietnam	Unpublished
^2^WEHI 155 SNPs	*P. falciparum*	155 SNPs all neutral variants, a subset are geographic markers	Infection complexity, genetic relatedness, geographic region	Fluidigm SNPType™ Assay	Papua New Guinea, Solomon Islands, Mali	Unpublished
Broad Institute 24 SNPs	*P. falciparum*	24 polymorphic, neutral variants	Infection complexity, genetic relatedness	High-resolution melt	Ethiopia, Malawi, Nigeria, Panama, Senegal, Zambia, Zimbabwe	[116]
^3^Swiss TPH 5 genes	*P. falciparum*	5 gene regions: *cpmp, cpp*, *ama1-D3, csp, msp7*	High sensitivity assessment of infection complexity, longitudinal tracking	Illumina amplicon sequencing	Papua New Guinea, Cambodia	[42]
^4^UCSF 100 microhaplotypes	*P. falciparum*	100 globally diverse multi-allelic amplicons	Infection complexity, genetic relatedness	Illumina amplicon sequencing	Mozambique	[117]
Broad Institute 42 SNPs	*P. vivax*	^5^42 polymorphic, neutral variants, including region-specific variants for geographic assessment	Infection complexity, genetic relatedness, geographic region	High-resolution melt	Brazil, French Guiana, Ethiopia, Sri Lanka (and countries below)	[116]
^1^SpotMalaria 113 SNPs	*P. vivax*	^5^38 of 42 Broad SNPs, plus species-specific variants, drug-resistance markers in *pvmdr1, pvdhfr, pvdhps*	Drug resistance prevalence, species confirmation, infection complexity, genetic relatedness, geographic region	Illumina amplicon sequencing	Afghanistan, Bangladesh, Bhutan, Cambodia, Colombia, Ethiopia, Indonesia, Iran, Mauritania, Malaysia, Myanmar, Republic of Korea, Sudan, Thailand, Vietnam	Unpublished
^2^WEHI 148 SNPs	*P. vivax*	148 SNPs, all neutral variants	Infection complexity, genetic relatedness	Illumina amplicon sequencing	Papua New Guinea, Cambodia	Unpublished
Menzies-Sanger 100 microhaplotypes	*P. vivax*	100 globally diverse multi-allelic amplicons	Infection complexity, genetic relatedness	Illumina amplicon sequencing	(Under development)	Unpublished

^1^https://www.malariagen.net/projects/spotmalaria. ^2^Walter and Eliza Hall Institute of Medical Research. ^3^Swiss Tropical and Public Health Institute. ^4^University of California, San Francisco. ^5^Barcodes designed with intentional overlap to support data sharing between partners

Building in-country processing capacity is a primary objective of the SpotMalaria and GenRe-Mekong genetic surveillance projects. Implementing high-throughput pipelines for genetic surveillance promotes ownership of the process, extends capacity in countries, and potentially delivers more timely products. To facilitate in-country implementations, the genotyping framework has been engineered using an amplicon sequencing approach, so that it can operate on lower-end Illumina sequencers and be deployed in malaria-endemic countries. Technology transfer is currently at an advanced stage in Ghana, the Gambia, Indonesia and Vietnam. Miotto, Nguyen Thuy-Nhien and Noviyanti described their respective experiences of the pros and cons of amplicon sequencing in malaria-endemic countries. In most cases, the adoption process has been gradual, owing to challenges such as infrastructure stability (such as electrical power), procuring specialized reagents, and the higher local costs of equipment, reagents and maintenance. Several endemic countries are not ready to make such a transition, so it is essential that they be guaranteed equal access to technology. For this reason, SpotMalaria and GenRe-Mekong have continued offering processing capacity at the Wellcome Sanger Institute, while encouraging labs in endemic countries to provide regional processing support for neighbouring countries.

Alyssa Barry gave an overview of Oxford Nanopore’s minION platform, which supports rapid data delivery, potentially within as little as 24 h of sample processing. The minION is a highly portable sequencer, which can be deployed in low-resource settings, with a lower start-up investment than Illumina sequencers but also lower throughput, which makes it more suitable for processing small sets of samples. It is also technically simpler than Illumina, requiring less specialized skills. This platform can be used for targeted genotyping of SNP barcodes, as well as sequencing of large and highly repetitive genomic regions. The flexibility to sequence entire regions in a non-targeted manner has potential for detecting new mutations, but it may be difficult for health workers or NMCPs to derive useful interpretations on these mutations. The main technical disadvantage of the minION is its sequencing error rate, higher than that of Illumina. The high error rate needs correcting by producing multiple reads covering the locus of interest and applying appropriate statistical methods.

**Fig. 2:**
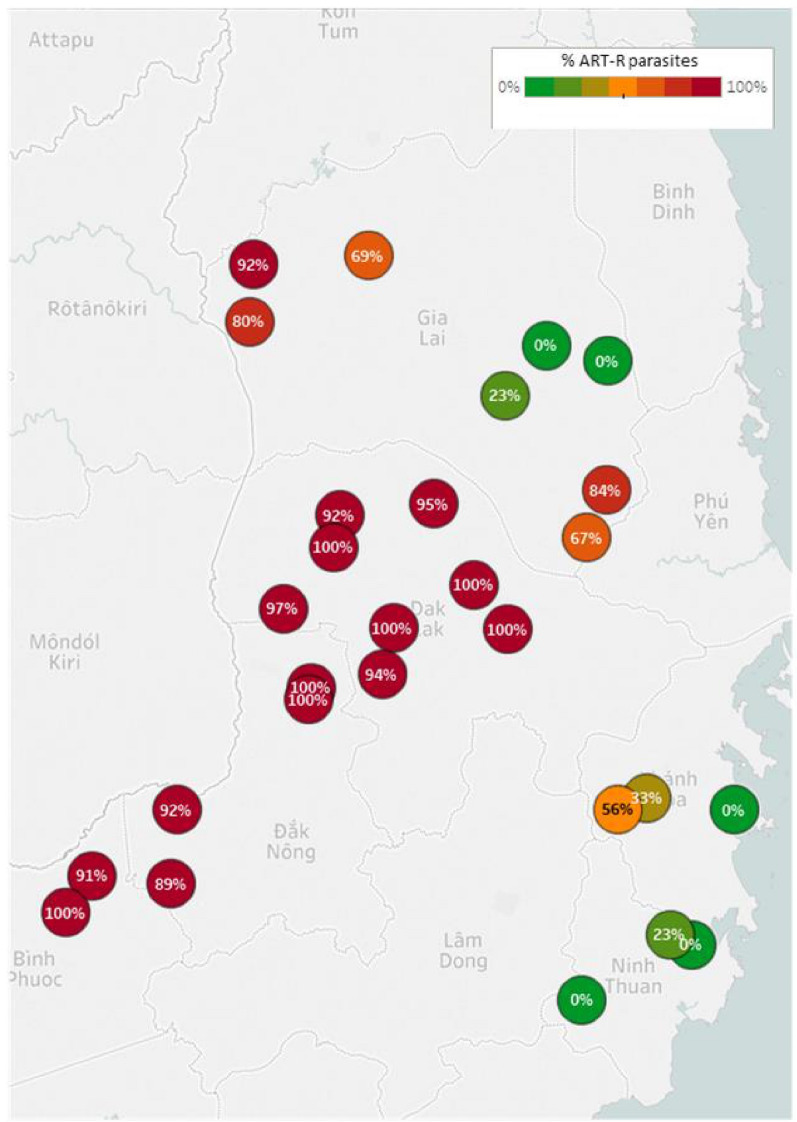
Example of a GenRe-Mekong drug resistance prevalence “traffic light” plot. Example of a map generated by the SpotMalaria GenRe-Mekong project, showing the predicted prevalence of artemisinin-resistant parasites at sites in 6 provinces of Vietnam before September 2018. District-level drug resistance-associated allele frequencies are coloured according to prevalence (ranging from 0–100% in a spectrum from green to red). Courtesy of the Institute of Malariology, Parasitology and Entomology, Quy Nhon and OUCRU, Vietnam

In addition to the laboratory aspects of these genotyping platforms, there were active discussions around the processing and interpretation of the data they produce. The partners agreed that both the Illumina and minION platforms present considerable bioinformatics challenges, highlighting the need for local capacity building to support data handling and analysis. Some participants identified data processing as a suitable task for service provision and placed greater importance on data interpretation and the need for user-friendly data outputs that can be readily used by control programme workers who may have limited knowledge of genetics. Data analysis tools are being developed and are building capacity to address the knowledge gaps between researchers and control programmes. However, these tools are in their infancy and further optimization and development is needed. The SpotMalaria team have developed an informatics pipeline to support the analysis of amplicon sequencing data uploaded from the Illumina sequencer, producing Genetic Report Cards, detailing individual-level information on the presence of mutations that confer resistance to a variety anti-malarial drugs in each infection (https://www.malariagen.net/projects/spotmalaria). In addition, GenRe-Mekong delivers reports containing maps of population-level drug resistance prevalence for different drugs, shown using intuitive “traffic light” colour representations (example in Fig. 2). Whilst the importance of user-friendly tools for the NMCPs was widely agreed on, there was also a keen interest in strengthening the local research capacity. The malaria research outputs from Eijkman provide inspiring examples of the potential for developing local bioinformatics expertise in the region. Collaborative projects between Eijkman and Menzies have established web-based, open access genetic data analysis tools such as vivaxGEN-GEO [68], vivaxGEN-MS [80], and vivaxGEN-SNP (in development). The vivaxGEN-MS platform currently hosts data from 11 countries and has supported data analysis for 10 publications to date. Enhancing local research in genetics and genomics will enable countries to implement molecular surveillance tools and tailored them to specific local needs.

### Data sharing within and between countries

The workshop discussed the relevance of data sharing to both researchers and NMCPs, along with the challenges that needed to be addressed. The consensus was that data sharing between institutions and across national borders is crucial for addressing use cases such as drug resistance gene flow, connectivity between populations, and border and imported malaria. There were no anticipated political obstacles to data sharing within countries. However, some countries perceive intellectual property as a potential challenge in sharing data. In other countries, such as Indonesia, national export restrictions constrain sharing of the raw sequence reads generated by whole genome sequencing. Such restrictions challenge open access but can be mitigated by sharing “intermediate datasets” such as the genotype calls used in whole genome sequencing studies, which ensures that the results are reproducible (e.g. [81] provides genotype calls in a *vcf* file format). Furthermore, this ensures that genotyping data produced at microsatellites or SNP-based barcodes can be made open access. The value of converging on commonly structured datasets was also discussed; for example, whether the broad range of SNP barcodes available for different use cases would be a challenge in sharing data between sites where there is little or no overlap in the SNP panels. The participants acknowledged that markers tailored to a specific country or region may have higher resolution than global marker sets to resolve the local genetic microepidemiology; however, they agreed that consensus was needed to ensure that at least part of the data generated in each country could be shared with others to support regional surveillance. For use cases such as assessment of transmission intensity, consensus markers would strengthen comparability between sites, but are not essential. However, for use cases that aim to assess the connectivity or drug resistance gene flow between populations, overseas importation, or cross-border transmission, a consensus marker set is critical. In addition, platforms and tools to support data sharing between partners within and across borders can overcome inability to share data due to a lack of mechanisms to do so effectively. Networks such as APMEN, ACREME, vivaxGEN, the Worldwide Antimalarial Resistance Network (WWARN) and MalariaGEN are important forums to support consensus and data sharing between countries. The APMEN vivax working group has been successful in establishing consensus for microsatellite-based genotyping markers across the network, when this technology was still the favoured genotyping method for *P. vivax* [80]. The network has also helped establish a platform supporting open-access sharing of the microsatellite-based genotyping data [80]. For SNP-based data, the platform underpinning GenRe-Mekong and SpotMalaria automatically aggregates data, presenting regional and global views. Data sharing can be integrated into the processing pipelines and thus come at no extra effort for the country. Ownership of the data is retained by the country, and adequate recognition and visibilities are given to the contributing party when the data are aggregated.

Ethical aspects of genetic surveillance were raised; specifically, it was highlighted that patient informed consent and assent can add a large additional work-load on top of routine tasks. Furthermore, the primary goal of surveillance outputs is to inform public health rather than to conduct research. In the Lao People’s Democratic Republic and Thailand, NMCPs are considering requesting an exemption from collecting additional patient consent, justified by the integration of sample collection in the standard treatment procedures, and the public health benefits of the resulting outputs. A critical condition for such exemption is that no human genetic data is collected. Different solutions will have to be adopted by countries that deem it important to genotype human genetic variants, for example to establish the prevalence of *glucose-6-phosphate dehydrogenase* (*G6PD*) variants or *cytochrome P450 2D6* (*CYP2D6*) variants that have implications for the safe and effective administration of primaquine [82].

### Maximizing the value of parasite genotyping for control programmes

The workshop ended with a session on maximizing the value proposition of molecular surveillance for malaria control programmes. Miotto highlighted that “it’s hard to appreciate the value of something you don’t have, and hard to predict what you will use in the future”. The full value and potential of genotyping tools may only be realized once they are actively implemented within surveillance frameworks on a wider scale. In order to reach that stage, the research community needs to ensure that they gain a good understanding of NMCPs activities, to ensure the molecular applications they develop serve these needs optimally. As emphasized by the Indonesian NMCPs, regular engagement between local researchers and NMCPs will be critical to achieve this. Ferdinand Laihad and Endang Sumiwi, from the Indonesian NMCP, emphasized the value of bringing together participants from different countries to share their local knowledge and experiences in malaria control and elimination.

There was active discussion concerning the financing of comprehensive surveillance frameworks. To achieve the routine and widespread sampling and processing needed for an informative surveillance operation, a stable source of funding is needed from local governments, the WHO, or other international funding agencies. Inclusion of genetic epidemiology in WHO policy will be needed to leverage funds from the Global Fund. It was widely agreed that more evidence of policy changes, as observed in Vietnam, and demonstration of the advantages and potential cost-saving of genetic surveillance tools are needed to encourage increases in funding from local governments and other key stakeholders. Large-scale evaluations of the cost-effectiveness of different use cases will be invaluable.

## Conclusion

The elimination of malaria from the Asia–Pacific region by 2030 presents a spectrum of formidable challenges and innovative approaches both within and between countries are needed. Molecular surveillance offers great potential to generate knowledge that will allow NMCPs to keep ahead of the constantly changing and evolving dynamics of parasite populations. Considerable work is still needed to support the implementation of genomic technologies and bioinformatics capacity in the region. Further research is needed to optimize methodologies for several use cases, and to build the evidence base on the utility and cost-efficacy of genetic epidemiology, to help leverage funding and logistic support for wide-scale genetic and genomic surveillance in-country. Engagement between NMCPs and researchers will be critical at each step, to ensure that these efforts meet the operational needs of malaria elimination.

## Abbreviations

ACREME: Australian Centre of Research Excellence in Malaria Elimination
ACT: Artemisinin-based Combination Therapy
APLMA: Asia Pacific Leaders Malaria Alliance
APMEN: Asia Pacific Malaria Elimination Network
CNVs: Copy Number Variants
COI: Complexity of Infection
CYP2D6: Cytochrome P450 2D6
DFAT: Department of Foreign Affairs and Trade
G6PD: Glucose-6-phosphate dehydrogenase
*k13*: *kelch-13*
*F*_ST_: Fixation index
*F*_WS_: Within-sample Fixation index
GMS: Greater Mekong Subregion
HRP2: Histidine-rich protein 2
HRP3: Histidine-rich protein 3
IBD: Identity By Descent
NMCP: National Malaria Control Programme
malariaGEN: Malaria Genomic Epidemiology Network
MOI: Multiplicity Of Infection
MORU: Mahidol Oxford Research Unit
OUCRU: Oxford University Clinical Research Unit
PCR: Polymerase Chain Reaction
*pfmdr1*: *P. falciparum multidrug resistance 1*
*pfpm2/pfpm3*: *P. falciparum plasmepsin 2/3*
PNG: Papua New Guinea
*pvcrt-o*: *P. vivax chloroquine resistance transporter*
*pvmdr1*: *P. vivax multidrug resistance 1*
RDTs: Rapid diagnostic tests
SNPs: Single Nucleotide Polymorphisms
SP: Sulfadoxine-pyrimethamine
STRIVE: Stronger Surveillance and Systems Support for Rapid Identification and Containment of Resurgent or Resistant Vector Borne Pathogens
sWGA: Selective whole genome amplification
UNICEF: United Nations Children's Fund
vivaxGEN: Vivax Genomic Epidemiology Network
WHO: World Health Organization
WWARN: Worldwide Antimalarial Resistance Network
